# Effects of Entomopathogenic Fungi on Individuals as Well as Groups of Workers and Immatures of *Atta sexdens rubropilosa* Leaf-Cutting Ants

**DOI:** 10.3390/insects12010010

**Published:** 2020-12-25

**Authors:** Luis Eduardo Pontes Stefanelli, Tarcísio Marcos Macedo Mota Filho, Roberto da Silva Camargo, Carlos Alberto Oliveira de Matos, Luiz Carlos Forti

**Affiliations:** 1 Laboratório de Insetos Sociais-Praga, Departamento de Produção Vegetal, Faculdade de Ciências Agronômicas, UNESP, Caixa Postal 237, Botucatu, SP 18603-970, Brazil; luis.stefanelli@unesp.br (L.E.P.S.); camargobotucatu@yahoo.com.br (R.d.S.C.); luiz.forti@unesp.br (L.C.F.); 2 Campus Experimental de Itapeva, UNESP, Itapeva, SP 18409-010, Brazil; carlos@itapeva.unesp.br

**Keywords:** *Beauveria bassiana*, Formicidae, leaf-cutting ants, sulfluramid, *Trichoderma harzianum*

## Abstract

**Simple Summary:**

The used active ingredient sulfluramid for toxic baits for the control of leaf-cutting ants has been included in Annex B of the Stockholm Convention on Persistent Organic Pollutants. The use of entomopathogenic fungi to control these insects has shown promising results, *Trichoderma harzianum* showed high pathogenicity against *A. sexdens rubropilosa* larvae and pupae, leading to a faster mortality and a decrease in survival rates. *Beauveria bassiana* was responsible for causing faster worker mortality and lower survival rates. An individual contaminated with *B. bassiana* or *T. harzianum* in a group decreases its survival rate, supporting the hypothesis that entomopathogenic fungi are efficient in controlling leaf-cutting ants when contaminated workers are allocated to groups of healthy workers.

**Abstract:**

In 2009, sulfluramid, the main ingredient in toxic baits for leaf-cutting ant control, was included in Annex B of the Stockholm Convention on Persistent Organic Pollutants. This resulted in interest in the use of entomopathogenic fungi such as *Beauveria bassiana* and *Trichoderma harzianum* for leaf-cutting ant control. The efficiency of these fungi in controlling these insects and the way that ants react individually or in group to the biological risks posed by these fungi is poorly understood. For this reason, we assessed the effects of *B. bassiana* and *T. harzianum* on *Atta sexdens rubropilosa* larvae, pupae and workers. Moreover, we investigated whether the number of contaminated individuals within a group has an influence in controlling the spread of fungi among workers. We found that the fungus *T. harzianum* showed high pathogenicity against *A. sexdens rubropilosa* larvae and pupae, leading to faster mortality and a survival rates. On the other hand, the fungus *B. bassiana* was responsible for causing faster worker mortality and lower survival rates. In addition, we observed that an increase in individuals contaminated with *B. bassiana* or *T. harzianum* in the group decreases its survival rate. The results support the hypothesis that entomopathogenic fungi are efficient in controlling leaf-cutting ants when contaminated workers are allocated to groups of healthy workers.

## 1. Introduction

Leaf-cutting ants of the genus *Atta* Fabricius 1805 and *Acromyrmex* Mayr 1865 (Hymenoptera: Formicidae) are eusocial insects exclusively found in Neotropical [1]. Growing the fungus *Leucocoprinus gongylophorus* (Heim, 1957), they feed on several species of plants of economic interest. They are known to be the main pests forest farming, agriculture and livestock [2,3]. 

Leaf-cutting ants are controlled with chemicals, especially those using toxic baits [3]. Those are the most low-cost and practical method available on the market [4]. In addition, they dispense with specialized manpower and equipment and facilitate the treatment of difficult to access nests [5]. They consist of a mixture of active ingredients that act by ingestion that are dissolved in soybean oil and incorporated into dehydrated citrus pulp pressed into pellets [6]. Sulfluramid 0.3% (*w*/*w*) is the only one that is efficient in controlling all species of leaf-cutting ants [3]. 

The production and the degradation of sulfluramid (EC/LIST n. 223-980-3; CAS n. 4151-50-2) through biological and abiotic mechanisms produces perfluorooctane sulfonate (PFOS), a highly persistent environmental contaminant [7,8]. PFOS has been associated with weight loss, reductions in serum cholesterol and in thyroid hormones, besides hepatotoxic and carcinogenic effects in humans and in some animals raised under laboratory conditions [9,10]. In 2009, sulfluramid was included in Annex B of the Stockholm Convention on Persistent Organic Pollutants, with its permission for use restricted to the control of leaf-cutting ants in Brazil until a new compound is found to replace it [11].

Biological control with the fungus *Beauveria bassiana* infecting workers in their colonies or with *Trichoderma harzianum* as an antagonist to symbiotic fungus has proven to be an efficient alternative to control leaf-cutting ants [12]. However, in the field, fungal action is limited by varied and efficient defense mechanisms on the part of ants [13]. Self-grooming, allogrooming (mutual grooming), nest cleaning and association with *Pseudonocardia* bacteria are all defense mechanisms that act by forming an immunological barrier of a social nature and inhibiting the action of parasites [14]. The metapleural gland of ants produces substances (indoleacetic acid (24–45%), palmitic acid (10–25%), 4-oxooctanoic acid (9–24%) and acetic acid (3–9%) valeric acid, hexanoic acid, heptanoic acid, octanoic acid, nonanoic acid, decanoic acid, palmitoleic acid, 2-nonanone, 2-nonanol, furfuryl alcohol, and indole) with antifungal and antibiotic action [15,16]. Allogrooming is effective in removing parasites such as *Metarhizium anisopliae* from their cuticles [17].

Moreover, the density of contaminated ants in a group of healthy ants influences the spread of pathogens within the colony, with the pathogen transmission rate being inversely proportional to the density of the healthy population [17]. Colonies of social insects have developed collective immune defenses against parasites. These “social immunity systems” result from the cooperation of individual group members to combat an increased risk of disease transmission resulting from sociality and living in groups [18].

Knowing the individual and group resistance mechanisms of leaf-cutting ants is essential when the intention is to implement safe and efficient methods for microbial control [19]. The present study assessed the pathogenicity of *B. bassiana* and *T. harzianum* on *Atta sexdens rubropilosa* immatures and workers under laboratory conditions. Additionally, we described the mortality rates of workers and immatures and their influence on controlling the spread of spores from entomopathogenic fungi among workers.

## 2. Materials and Methods 

### 2.1. Studied Colonies

Colonies of *Atta sexdens rubropilosa* Forel 1908 (Hymenoptera: Formicidae), approximately four months old, were collected in March 2020 in the municipality of Botucatu, São Paulo, Brazil. Subsequently, they were subjected to a temperature of 24 ± 2 °C, relative humidity of 80% and photoperiod of 12 h of light in the Laboratory of Social Insects-Pests (Laboratório de Insetos Sociais-Praga) (LISP) of the São Paulo State University’s School of Agronomic Sciences, until the bioassays started. 

The colonies were individually housed in 1.5 l acrylic plastic containers (GEP Comercial^TM^) whose bottoms were covered with a 1.0 cm plaster layer to keep the fungus garden moist. The fungus garden container was connected to two equidistant 250 mL plastic containers: one for foraging of the plants supplied and the other for waste disposal. Leaves of the *Acalypha* spp. plant were provided every two days in the foraging container to maintain the growth of the symbiotic fungus.

### 2.2. Bioassay 1: Pathogenicity of Fungi against Immature and Adult Leaf-Cutting Ants

The pathogenicity of *B. bassiana* and *T. harzianum* was assessed in *A. sexdens rubropilosa* immatures and adults (workers with head length from 1.2 to 2.2 mm). To do so, a completely randomized design was used, with treatments consisting of three development stages (larva, pupa and adult) and four concentrations of two commercial products owned by the company Koppert Biological Systems^®^ City Piracicaba, Brazil: Boveril WP PL63^®^, composed of 5% of *Beauveria bassiana* (Bals.) Vuill., strain PL63 (minimum of 1.0 × 10^8^ viable conidia g^−1^), and Trichodermil SC 1306^®^, composed of 4.8% of *Trichoderma harzianum* Rifai, strain ESALQ-1306 (minimum of 2.0 × 10^9^ viable conidia mL^−1^). 

The individuals were collected from the laboratory colonies flexible aluminum entomological forceps (Log Nature^TM^). Afterwards, they were dipped for two seconds in suspensions of distilled water and 1% Tween 80^®^ with the following concentrations: 1.0 × 10^3^ (Bov3), 1.0 × 10^4^ (Bov4), 1.0 × 10^5^ (Bov5) and 1.0 × 10^6^ (Bov6) conidia mL^−1^, for *B. bassiana*, and 2.0 × 10^4^ (Tri4), 2.0 × 10^5^ (Tri5), 2.0 × 10^6^ (Tri6) and 2.0 × 10^7^ (Tri7) conidia mL^−1^ for *T. harzianum*. There were two negative controls: (i) groups immersed in 1% Tween 80^®^ aqueous solution (Control 1) and (ii) groups immersed in distilled water (Control 2) (adapted methodology) [20,21]. The 1% Tween 80^®^ promotes a homogeneous suspension allowing uniform application of conidia in immature and adults. For each treatment, there were four repetitions (sterile Petri dishes), each containing groups of five individuals. We used concentrations according to experimental protocol developed by Loureiro and Monteiro [20,21].

**Figure 1:**
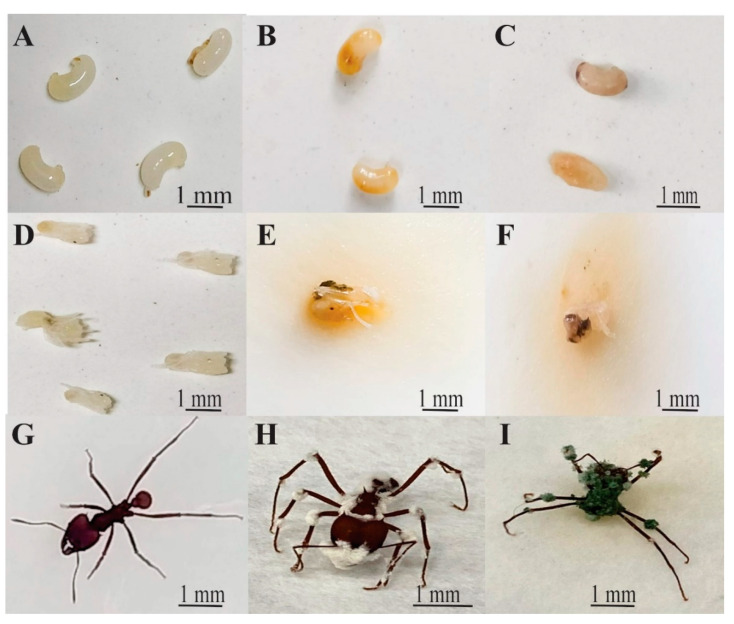
Stages of *Atta sexdens rubropilosa* Forel 1908 (Hymenoptera: Formicidae) exposed with the different entomopathogens under laboratory conditions: (**A**) healthy pupae; (**B**) pupae exposed with *Beauveria bassiana*; (**C**) pupae exposed with *Trichoderma harzianum*; (**D**) healthy larvae; (**E**) larvae exposed with *Beauveria bassiana*; (**F**) larvae exposed with *Trichoderma harzianum*; (**G**) healthy worker; (**H**) worker exposed with *Beauveria bassiana*; (**I**) worker exposed *Trichoderma harzianum*.

Subsequently, the groups were placed in sterile Petri dishes containing filter paper moistened with distilled water at the bottom and kept in a BOD incubator (Eletrolab^TM^), being subjected to a temperature of 27 ± 1 °C, relative humidity of 70 ± 10% and a 12-h photoperiode to maintain the optimal conditions for the development of the fungi and the insects (larvae, pupae and workers). During the bioassay, the workers were not provided with any food [20,21]. The assessments were carried out daily for five days, and the mortality of the individuals in each treatment was recorded. We observed the change of color in immatures and fungus colonizing the dead workers (Figure 1). 

### 2.3. Bioassay 2: Response of the Group of Workers to the Contamination of Colony Mates

Based on the experiment, the group of the spread occurring on a colony scale, treatments Bov6 and Tri7 were used, because they provided the shortest Lethal time of 50% of mortality (LT_50_). For each treatment being studied, the workers were divided into groups, and each group was composed of five repetitions, arranged as follows:

(1) Group 1:19—1 contaminated worker + 19 healthy workers = 20 workers
(2) Group 2:18—2 contaminated workers + 18 healthy workers = 20 workers
(3) Group 4:16—4 contaminated workers + 16 healthy workers = 20 workers
(4) Group 8:12—8 contaminated workers + 12 healthy workers = 20 workers
(5) Group 16:4—16 contaminated workers + 4 healthy workers = 20 workers
(6) Group 18:2—18 contaminated workers + 2 healthy workers = 20 workers
(7) Group 19:1—19 contaminated workers + 1 healthy worker = 20 workers

The groups were transferred to acrylic pots measuring 7.5 cm in diameter and 5.5 cm in height, with hermetic lids, containing a 1.0 cm plaster layer at the bottom and a small amount (3.0 g) of the symbiotic fungus that belonged to the colony from which the workers were removed. Subsequently, individuals were marked with a white-colored pen (Edding^®^, Ahrensburg, Germany) and later contaminated by dipping into spore suspension for 10 s. This pen was used due to its excellent adhesion, quick drying and good visibility. This technique has been widely used for leaf-cutting ants, the dried ink did not impede the movement of the ants at all [22]. The marking was done in order to distinguish contaminated individuals from healthy ones. The assessments were conducted daily for five days, and the mortality of contaminated and non-contaminated individuals was recorded according to Figure 1.

### 2.4. Data Analysis

The lethal time (LT_50_) to cause 50.0% of mortality in *A. sexdens rubropilosa* immatures and adults was obtained by the PROBIT analysis (Finney, 1971), using SAS [23]. 

The Kaplan-Meier estimator (also known as the product-limit estimator) was used to calculate the survival function [24]. This estimator is an adaptation of this empirical survival function:

$$\hat{S}\;\left(t\right)\;=\;\frac{Number\;of\;individuals\;that\;survived\;until\;time\;t}{Number\;total\;of\;individual\;in\;the\;study}$$

This function implies the absence of censorships, and presence of incomplete or partial information [25]. $\hat{S}\left(t\right)$ is a staircase function with steps that inform the time at which the individual’s death occurred. The size of the steps is 1/n (n = sample size), which is multiplied by the number of ties in case they occur.

The Log-rank, or Mantel Haenszel, test was employed to test the hypothesis of non-existence of differences in the survival functions between treatments. The *p* values were adjusted by Benjamini and Hochberg’s method [26], which controls the false discovery rate (the expected proportion of false discoveries among rejected hypotheses).

The ggplot2, survival and survminer packages of the R software, version 4.0.0, were used for statistical computing and graphing [27]. 

## 3. Results

### 3.1. Bioassay 1: Pathogenicity of Fungi against Immature and Adult Leaf-Cutting Ants

Based on the lethal times (LT_50_), the entomopathogenic activity of the fungi *B. bassiana* and *T. harzianum* was observed for *A. sexdens rubropilosa* workers, and larvae and pupae (Table 1 and Figure 1). Overall—for both development stages are inversely proportional to the concentrations of conidia in the solutions. For the larva and pupa stages, *T. harzianum* expressed greater pathogenicity for workers with the LT_50_ varying from 25.251 (Tri4) to 7.385 (Tri7) hours (Table 1), and from 28.602 (Tri4) to 11.503 (Tri7) hours for larvae and pupae, respectively. On the other hand, *B. bassiana* expressed greater pathogenicity for workers, with the LT_50_ varying between 125.746 (Bov3) and 9.949 (Bov6) hours. Broadly speaking, the adult stage was less susceptible to the action of the fungi being studied.

**Figure 2:**
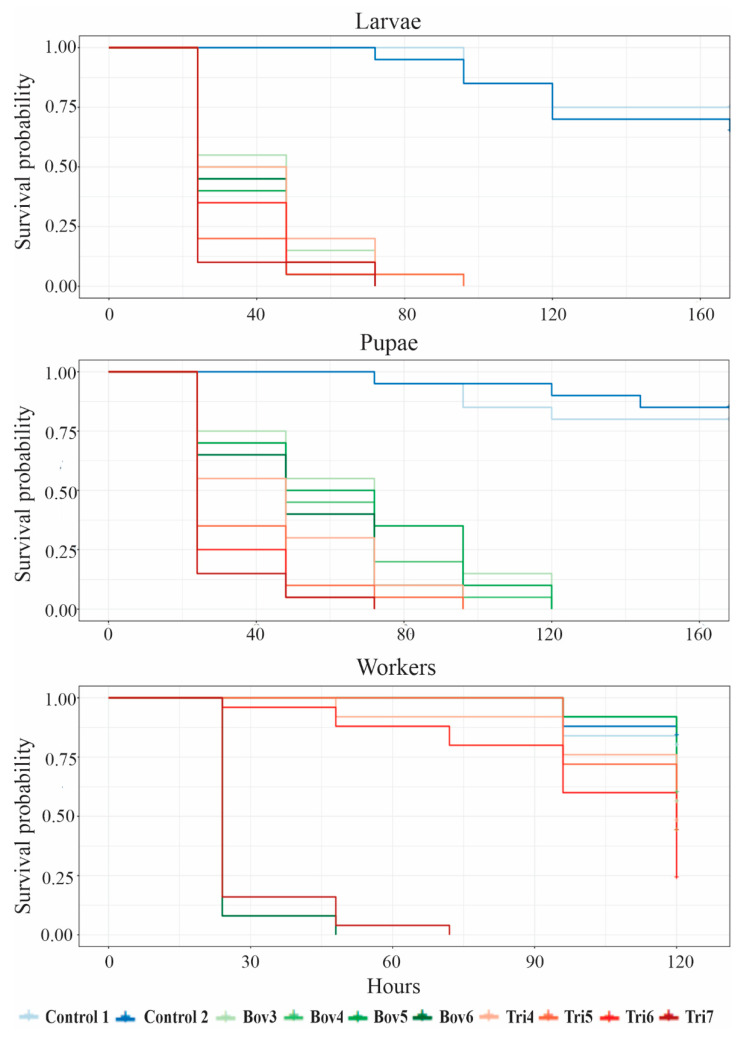
Survival curve of *Atta sexdens rubropilosa* Forel 1908 (Hymenoptera: Formicidae) larvae, pupae and workers immersed in suspensions of *Beauveria bassiana* and *Trichoderma harzianum*.

The survival curves of *A. sexdens rubropilosa* larvae at different concentrations of *B. bassiana* and *T. harzianum* conidia showed no significant between them, but were significantly different from the curves of the negative controls, Control 1 and Control 2 (Table 2 and Figure 2). 

For pupae, overall, there was a tendency to survival differences between the different concentrations of *T. harzianum* and *B. bassiana* (Table 2 and Figure 2), with the differences between the concentrations with *T. harzianum* being lower.

In workers, survival at the Bov6 concentration inferior to that at the other concentrations of *B. bassiana* and *T. harzianum* (Table 2 and Figure 2). Survival at the Tri6 concentration does not differ only from survival at Tri4 and Tri5. Survival at the Tri7 concentration, in its turn, differs from all other concentrations.

### 3.2. Bioassay 2: Response of the Group of Workers to the Contamination of Colony Mates

**Figure 3:**
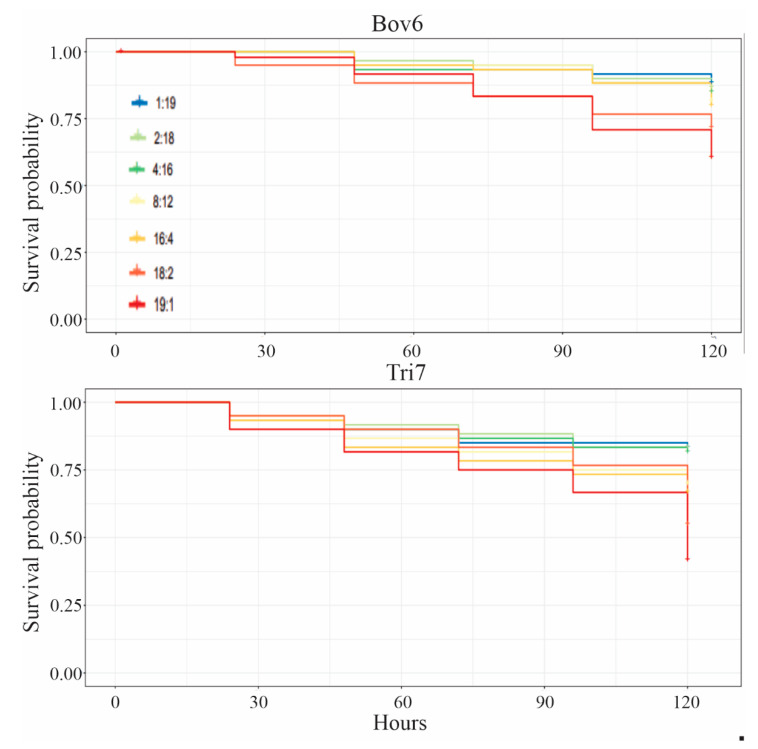
Survival curve of groups of *Atta sexdens rubropilosa* Forel 1908 (Hymenoptera: Formicidae) workers immersed in suspensions of *Beauveria bassiana* (Bov6) and *Trichoderma harzianum* (Tri7).

Concerning the groups contaminated with *B. bassiana*, the survival of the 19:1 group differed significantly from that of the 1:19, 2:18 and 4:16 groups (Table 3 and Figure 1 and Figure 3). Comparing the survival of the *T. harzianum* groups, the 18:2 group differed from the 1:19, 2:18 and 4:16 groups. As for the 19:1 group, it differed from the 1:19, 2:18, 4:16, 8:12 and 16:4 groups. Thus, it can be inferred that the increase in individuals contaminated with *B. bassiana* or *T. harzianum* in the group results in lower survival rates for the whole.

## 4. Discussion

### 4.1. Bioassay 1: Pathogenicity of Fungi against Immature and Adult Leaf-Cutting Ants

Our present study, conducted under laboratory conditions, showed that the treatments containing *B. bassiana* and *T. harzianum* conidia were efficient in controlling *A. sexdens rubropilosa*, as they caused the death of larvae, pupae and workers. It was interesting that all dead insects showed mycosis in their tegument, as present in Figure 1. *B. bassiana* has a biological cycle of approximately 168 h [28], and *T. harzianum*, of 120 h nder laboratory condition [29]. The death of the development stages is probably associated with toxic secondary metabolites for the insects synthesized by these fungi after the latter penetrates into the exoskeleton of the former [30].

*Beauveria* species are known to produce secondary metabolites with insecticidal properties, such as beauvericin [31], bassianolide [32] and bassiacridin [33]. For instance, bassianolide was toxic to *Bombyx mori* L. (Lepidoptera: Bombycidae) when incorporated into their diet or injected into the larvae [32]. Beauvericin showed insecticidal activity against *Calliphora erythrocephala* (Diptera: Calliphoridae), *Aedes aegypti* (Diptera: Culicidae) and *Spodoptera frugiperda* (Lepidoptera: Noctuidae) [34,35]. *Trichoderma harzianum*, despite not being an entomopathogenic fungus, also has a toxic effect on insects, which is attributed to secondary metabolites synthesized after penetration into the exoskeleton. Studies report that metabolites not yet identified, produced by *T. harzianum*, are toxic to *Periplaneta americana* [36]. In addition, extracts from *Trichoderma* spp isolates produced secondary metabolites that presented toxicity against *A. sexdens rubropilosa*, via ingestion, contact or exposure to volatile metabolites [37].

The LT_50_ for larvae, pupae and workers obtained in this study were shorter (Table 1) than those found by Loureiro and Monteiro [21] for isolates JAB 06 and AM 9 of *B. bassiana*, in *A. sexdens sexdens* soldiers, with an LT_50_ of 2.60 and 2.72 days, observed for the doses of 1.0 × 10^9^ conidia mL^−1^ and 1.0 × 10^8^ conidia mL^−1^, respectively. Our LT_50_ results were also inferior compared to those reported by Loureiro and Monteiro [20], for *A. sexdens sexdens* workers, with isolates JAB 06 and AM 9 of *B. bassiana* providing an LT_50_ of 2.80 (1.0 × 10^9^ conidia mL^−1^) and 2.16 (1.0 × 10^9^ conidia mL^−1^) days, respectively. When it comes to the fungus *T. harzianaum*, the results showed inferior for larvae, pupae and workers compared to the results obtained by Mussi-Dias et al. [37], according to whom the isolate of *Trichoderma* spp. caused 50% of mortality for workers after 2, 1.5 and 4 days when ingested, sprayed on workers, or by exposure to volatiles, respectively. 

The survival rates of *A. sexdens rubropilosa* larvae, pupae and workers contaminated with *B. bassiana* and *T. harzianum* (Table 2, Figure 1) were inferior to those of *Atta bisphaerica* workers contaminated with 1 μL of *B. bassiana* suspension at concentrations (conidia mL^−1^) of 10^5^ (20 days), 10^6^ (20 days), 10^7^ (9 days), 10^8^ (6 days) and 10^9^ (6 days) [19]. The survival results for both stages were also inferior to those found by Dornelas et al. [38], according to whom, on the tenth assessment day, all *A. sexdens* workers contaminated with 1 μL (1.0 × 10^7^ conidia mL^−1^) of *Metarhizium anisopliae* suspension. 

The variation in the LT_50_ and survival rates of the development stages of *A. sexdens rubrobilosa* contaminated with the different fungi may be associated with the capacity of infection and penetration of the fungi, the susceptibility of the host, and the number of toxic metabolites produced by these fungi. Moreover, the genetic variability present in entomopathogenic fungi [39] may be one of the factors responsible for differences in virulence between isolates and species, as reported by Diehl-Fleig et al. [40] 

Our results evidence that the fungi *B. bassiana* and *T. harzianum* are promising for the prospection of biological-control products. Overall, for both fungi, the lethal times (LT_50_) and estimated survival obtained in this study were superior for workers than for larvae and pupae (Table 1 and Table 2, Figure 1 and Figure 2). Entomopathogenic fungi, as mentioned earlier, infect their host via penetration. Penetration can occur anywhere in the cuticle, although preferred sites have been observed in several insects [41]. However, development stages that present a more rigid cuticle, that is, a more sclerotized one, can hinder the action of these fungi [42]. This fact could explain the LT_50_ and survival rates of the workers, because their cuticle is totally sclerotized and provides protection against desiccation, parasitism and predation [43]. In the specific case of fungus-growing workers, when pathogens come into contact with the surface of their cuticle, these ants perform allogrooming [44], in addition to producing antifungal substances in their metapleural gland that inhibit the action of pathogens [15]. These defense mechanisms are not present in the larva and pupa stages.

### 4.2. Bioassay 2: Response of the Group of Workers to the Contamination of Colony Mates

In general, the number of workers contaminated with *B. bassiana* or *T. harzianum* influenced the survival of the whole groups, with greater survival being found in groups with a larger number of healthy workers (Table 3 and Figure 3). Similar results were found by Hughes et al. [17] who reported that one single worker contaminated with the fungus *Metarhizium anisopliae*, when in contact with a group of healthy workers, presented a greater survival in relation to the contaminated workers that stayed isolated. This greater survival is related to numerous defense mechanisms on the part of ants. 

Among these mechanisms, the following are worth highlighting: hygienic behaviors referred to as grooming (self-grooming and allogrooming) and weeding to remove the spores of entomopathogenic fungal and prevent garden infection [15]; management of waste produced to prevent the spread of potentially harmful microbes from the waste to the garden [45]; and a mutualistic association with filamentous bacteria (*Pseudonocardia*) housed in the cuticle of ants that produce antibiotics that inhibit entomopathogenic fungi [46].

Noteworthy as well, is the presence of the infrabuccal cavity, a filtration structure within the oral cavity of ants [47] in which potentially dangerous spores and scrap that workers accumulate while grooming themselves or weeding the fungus garden are stored [48], and which, once filled, compresses and expels the material from this cavity in the form of an infrabuccal pellet to waste piles away from their nest in order to prevent microorganisms from re-infecting the garden [49]; and the production of numerous substances by the metapleural gland that are capable of acting as a colony defense agent [50].

However, we can observe that in groups in which the number of contaminated workers is equal to or greater than four, the benefit of being in a group does not present advantages in controlling the spread of pathogens and, consequently, means lower survival rates for the group, as observed for the groups that had workers contaminated with *T. harzianum* (Table 3 and Figure 2). This is probably caused because the ants are contaminated with a large amount of fungal conidia and cannot efficiently control the spread of spores. In this context, allogrooming does not work anymore with increased infected individuals, this it was observed by Camargo et al. [51]. 

This information becomes essential for the control of fungus-growing ants by means of entomopathogenic fungi. Several investigations have attempted to adapt the use of entomopathogenic fungi to control leaf-cutting ants through granulated baits with attractive substrate [52,53,54]. Some are efficient in laboratory conditions but, in field conditions, they stumble upon the social immunity of ants. Because when baits with fungal spores are used, few workers have direct contact with the spores [55]. The other workers are contaminated through interactions between individuals, as proved by Camargo et al. [51], who used a tracer dye whose dissemination was attributed to contact between workers. However, when it comes to entomopathogenic fungi, this dissemination does not occur because ants recognize entomopathogenic agents and use individual and group defense mechanisms that inhibit fungal action [15]. Our results show that, for greater efficiency in biological control with entomopathogenic fungi, there must be a large number of contaminated workers and maximum contact between fungus and host.

## 5. Conclusions

*Atta sexdens rubropilosa* immatures and adults are susceptible to the entomopathogenic action of the fungi *B. bassiana* and *T. harzianum*. Life in a group influenced the survival of workers, with shorter survival being observed in groups with a greater number of contaminated individuals. 

## Figures and Tables

**Table 1:** Lethal times (LT_50_) in hours for the entomopathogenic activity of the fungi *Beauveria Bassiana* and *Trichoderma harzianum* against *Atta sexdens rubropilosa* Forel 1908 (Hymenoptera: Formicidae) larvae, pupae and adults (workers) (n = 20).

Treatments	Larvae				Pupae				Adults			
	LT_50_	95% CI	X^2^	*p*-Value	LT_50_	95% CI	X^2^	*p*-Value	LT_50_	95% CI	X^2^	*p*-Value
Bov3	26.184	17.57–32.741	0.679	0.878	45.581	34.662–55.663	5.4560	0.141	125.746	116.547–158.214	0.056	0.997
Bov4	23.479	14.619–29.762	1.483	0.686	37.854	28.268–46.196	3.0391	0.386	125.576	115.837–163.423	0.082	0.994
Bov5	21.491	11.663–27.629	0.951	0.813	41.710	30.585–51.501	5.8373	0.120	117.410	110.806–129.714	0.016	0.999
Bov6	22.870	14.551–28.002	0.201	0.977	33.641	25.116–40.931	4.0577	0.255	9.949	0.00049–18.707	0.739	0.864
Tri4	25.251	15.755–32.280	1.322	0.724	28.602	18.930–36.142	3.043	0.385	129.563	107.324–204.560	2.980	0.395
Tri5	21.088	10.072–28.675	1.133	0.769	18.630	6.930–26.471	0.896	0.826	113.733	105.355–129.468	1.575	0.665
Tri6	20.040	9.006–25.808	0.299	0.960	16.450	2.848–23.398	0.452	0.929	99.269	84.208–127.522	5.329	0.149
Tri7	7.385	0.000–18.387	2.751	0.432	11.503	0.0026–20.453	0.723	0.868	12.431	0.307–20.157	0.585	0.899

CI = Confidence interval at 95% probability.

**Table 2:** *p* value of the survival analysis for *Atta sexdens rubropilosa* Forel 1908 (Hymenoptera: Formicidae) larvae, pupae and adults (workers).

Treat	Stage	Control 1	Control 2	Bov3	Bov4	Bov5	Bov6	Tri4	Tri5	Tri6
Control 2	Larvae	0.690	-	-	-	-	-	-	-	-
	Pupae	0.670	-	-	-	-	-	-	-	-
	Adults	0.764	-	-	-	-	-	-	-	-
Bov3	Larvae	**4.16 × 10^−4^**	**6.41 × 10^−4^**	-	-	-	-	-	-	-
	Pupae	**4.90 × 10^−6^**	**3.32 × 10^−4^**	-	-	-	-	-	-	-
	Adults	0.180	0.089	-	-	-	-	-	-	-
Bov4	Larvae	**9.55 × 10^−4^**	**1.02 × 10^−5^**	0.690	-	-	-	-	-	-
	Pupae	**7.85 × 10^−4^**	**1.48 × 10^−5^**	0.330	-	-	-	-	-	-
	Adults	0.262	0.136	0.820	-	-	-	-	-	-
Bov5	Larvae	**3.61 × 10^−4^**	**3.61 × 10^−4^**	0.448	0.690	-	-	-	-	-
	Pupae	**3.37 × 10^−5^**	**2.98 × 10^−5^**	0.735	0.486	-	-	-	-	-
	Adults	**0.048**	**0.019**	0.500	0.363	-	-	-	-	-
Bov6	Larvae	**3.61 × 10^−4^**	**4.16 × 10^−4^**	0.448	0.690	0.982	-	-	-	-
	Pupae	**3.32 × 10^−5^**	**1.38 × 10^−5^**	0.087	0.468	0.154	-	-	-	-
	Adults	**7.5 × 10^−13^**	**7.5 × 10^−13^**	**7.5 × 10^−13^**	**7.5 × 10^−13^**	**7.5 × 10^−13^**	-	-	-	-
Tri4	Larvae	**3.61 × 10^−4^**	**5.81 × 10^−4^**	0.991	0.690	0.448	0.448	-	-	-
	Pupae	**1.99 × 10^−5^**	**7.39 × 10^−4^**	0.042	0.254	0.078	0.631	-	-	-
	Adults	**0.044**	**0.019**	0.421	0.322	0.820	**2.8 × 10^−12^**	-	-	-
Tri5	Larvae	**3.61 × 10^−4^**	**3.61 × 10^−4^**	0.321	0.381	0.690	0.690	0.374	-	-
	Pupae	**6.49 × 10^−4^**	**3.17 × 10^−4^**	**0.003**	**0.019**	**0.006**	0.064	0.120	-	-
	Adults	**0.026**	**0.010**	0.302	0.217	0.640	**7.5 × 10^−13^**	0.835	-	-
Tri6	Larvae	**3.61 × 10^−4^**	**3.61 × 10^−4^**	0.302	0.448	0.690	0.690	0.310	0.919	-
	Pupae	**3.17 × 10^−4^**	**3.17 × 10^−4^**	**0.001**	**0.002**	**0.001**	**0.005**	**0.028**	0.414	-
	Adults	**0.001**	**9.1 × 10^−5^**	**0.010**	**0.006**	**0.037**	**2.2 × 10^−11^**	0.107	0.136	-
Tri7	Larvae	**3.61 × 10^−4^**	**4.16 × 10^−4^**	0.084	0.130	0.416	0.366	0.119	0.690	0.603
	Pupae	**3.17 × 10^−4^**	**3.17 × 10^−4^**	**9.56 × 10^−9^**	**0.001**	**0.001**	**0.002**	**0.012**	0.211	0.631
	Adults	**3.7 × 10^−13^**	**3.7 × 10^−13^**	**3.7 × 10^−13^**	**3.7 × 10^−13^**	**3.7 × 10^−13^**	0.349	**2.1 × 10^−12^**	**3.7 × 10^−13^**	**3.7 × 10^−11^**

* *p*-values significant in bold (*p* < 0.05).

**Table 3:** *p* value of the survival analysis for groups of *Atta sexdens rubropilosa* Forel 1908 (Hymenoptera: Formicidae) workers contaminated with *Beauveria bassiana* (Bov6) and *Trichoderma harzianum* (Tri7).

Group	Treat	1:19	2:18	4:16	8:12	16:4	18:2
2:18	Bov6	0.820	-	-	-	-	-
	Tri7	0.977	-	-	-	-	-
4:16	Bov6	0.727	0.820	-	-	-	-
	Tri7	0.865	0.865	-	-	-	-
8:12	Bov6	0.517	0.661	0.766	-	-	-
	Tri7	0.165	0.165	0.216	-	-	-
16:4	Bov6	0.439	0.517	0.661	0.820	-	-
	Tri7	0.093	0.093	0.137	0.810	-	-
18:2	Bov6	0.074	0.111	0.182	0.364	0.439	-
	Tri7	**0.010**	**0.010**	**0.017**	0.236	0.433	-
19:1	Bov6	**0.016**	**0.018**	**0.029**	0.062	0.074	0.506
	Tri7	**0.0001**	**0.0001**	**0.0002**	**0.018**	**0.048**	0.181

* *p*-values significant in bold (*p* < 0.05).
